# Supplementary figures and images for: MiRNAs differentially expressed in vegetative and reproductive organs of Marchantia polymorpha – insights into their expression pattern, gene structures and function
Source: RNA Biol. 2024 Feb 1;21(1):1–12. doi: 10.1080/15476286.2024.2303555 (PMC10841014; doi:10.1080/15476286.2024.2303555)

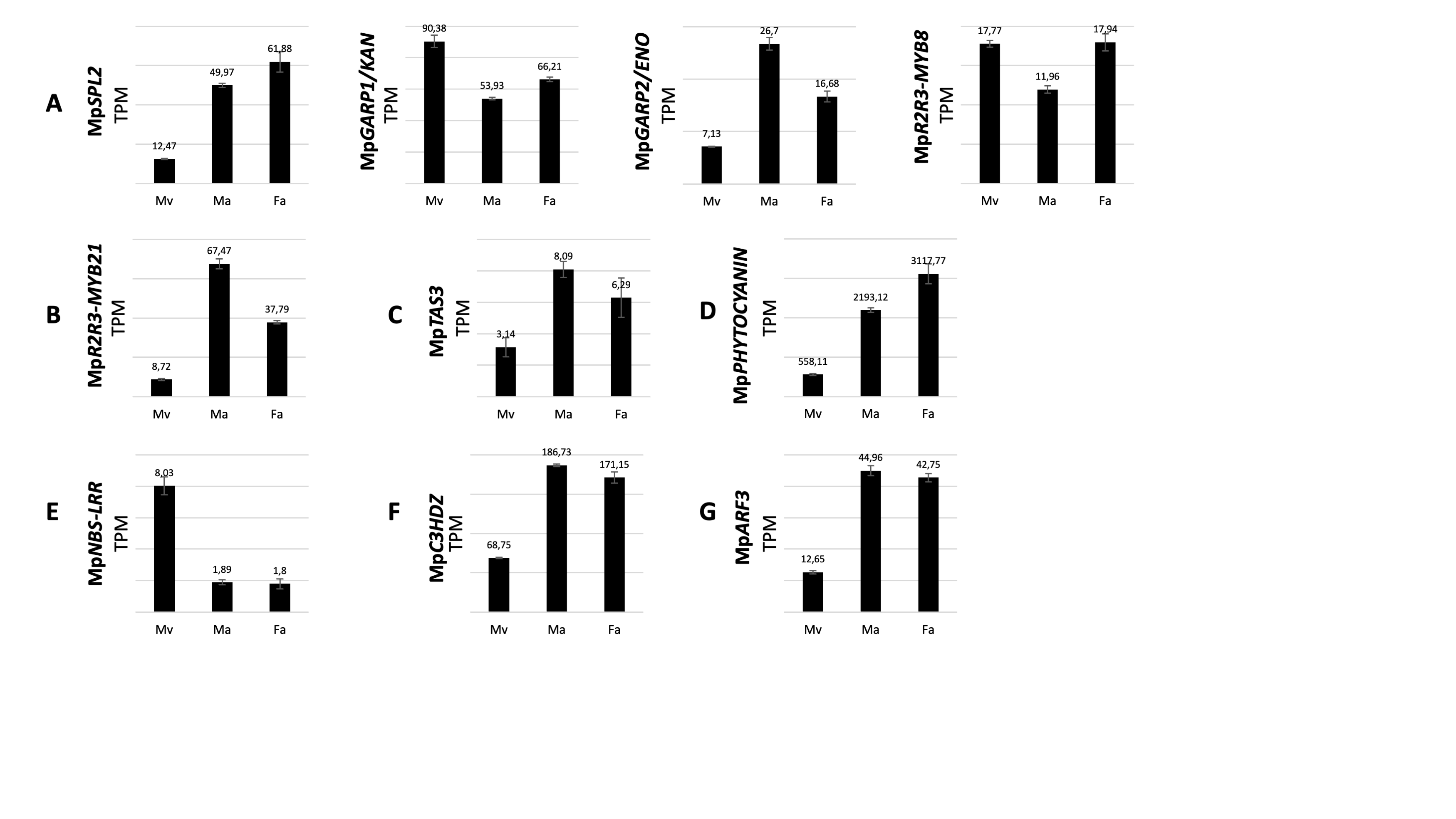

Supplement: S1.tiff [file KRNB_A_2303555_SM0154.tiff]
