# Supplementary material for: MiRNAs differentially expressed in vegetative and reproductive organs of Marchantia polymorpha – insights into their expression pattern, gene structures and function
Source: RNA Biol. 2024 Feb 1;21(1):1–12. doi: 10.1080/15476286.2024.2303555 (PMC10841014; doi:10.1080/15476286.2024.2303555)
Supplement: Table S1 .pdf [file KRNB_A_2303555_SM0153.pdf]

Supplementary Table1: List of primers used in the study

Name	Sequence (5'→3')	Use
5RLM_737a_R1	CAGGCCCATGGAAGGAAACA	5'-RLM RACE
5RLM_737a_R2	GTGACCACCGATCCATAGATG	5'-RLM RACE
3RACE_737a_R1	CATCTATGGATCGGTGGTCAC	3'-RACE
3RACE_737a_R2	GTAGACAATCTGCTTGGTCCG	3'-RACE
FLT_737a_F1	GTATACAGTCCTCGCTCTGA	full-length transcript
FLT_737a-F2	CAGTCCTCGCTCTGAAAAGCAC	full-length transcript
FLT_737a_R1	CTGCAAGCGATAATTCTTCCG	full-length transcript
FLT_737a_R2	ATACTATATCAAAAGTGCAGTAGAC	full-length transcript
FLT_11865_F1	ACTGGCCTGCCGCGATCGGA	full-length transcript
FLT_11865_F2	GGAGCGACCTGTTCGATCCTTTGC	full-length transcript
5RLM_11865_R1	GCAAGTGCAACTCCCAGCCAA	5'-RLM RACE
5RLM_11865_R2	GCACCTGCTGTGTGAAGCAT	5'-RLM RACE
3RACE_11865_R1	GGATTCTAGTCAGGGCACTCAT	3'-RACE
3RACE_11865_R2	CAATCTCGGGTACAGACCTT	3'-RACE
FLT_11865_R1	CGAGCCATTCACTTTGTAGAT	full-length transcript
FLT_11865_R2	CACGCGTTCGCATTTCTTAACATACG	full-length transcript
3RACE_11887_R1	AGTAAGTGCACGCGGCGACTT	3'-RACE
3RACE_11887_R2	GTATGCGTTTGTGTCTAGGC	3'-RACE
5RLM_11887_R1	GTCTTAGCTCCCTAACACTTTCC	5'-RLM RACE
5RLM_11887_R2	GCGACACGCGGTTAGTTATG	5'-RLM RACE
FLT_11887_F1	AGTACCCTTTCGATCGAGGTCA	full-length transcript
FLT_11887_R1	GTGCAGTTCTCCTTCAGTAGGAAGAG	full-length transcript
FLT_11887_R2	CACAGTAACTCGAGGAAGTAC	full-length transcript
5RLM_11796_R1	AAGGCCTCGTAAGCACACTCA	5'-RLM RACE
5RLM_11796_R2	CGAGGAGGCGCGTAGATCCCA	5'-RLM RACE
3RACE_11796_R1	GATTTGTGGGATCTACGCGCCT	3'-RACE
3RACE_11796_R2	AGATCCCACCGACCGCCTGAGT	3'-RACE
FLT_11796-F	TAAAGAGCAATGCCACTCTCGGG	full-length transcript
FLT_11796-R	GCAATTATGCAATGTTCTGTCTGC	full-length transcript
pri11737a-F	GATCGGTGGTCACGAAGCTT	RT-qPCR
pri11737a-R	AACCACTGGACGATGCATGA	RT-qPCR
pri11865-F	GCACTCATTATTGCTTTATC	RT-qPCR
pri11865-R	GCACACCATGGCCTTTGCGT	RT-qPCR
pri11796-F	AAGTCCTCTGAAGAACATCC	RT-qPCR
pri11796-R	AAGGCCTCGTAAGCACACTCA	RT-qPCR
pri11887-F	GGAAAGTGTTAGGGAGCTAA	RT-qPCR
pri11887-R	GGAAAACATTAGAGAGCTTAGC	RT-qPCR
pri11737b-F	ATGGAGCTCCGGACATTCAT	RT-qPCR
pri11737b-R	ATGGAGCACGACAAGCATAT	RT-qPCR
MpACT-F	AGGCATCTGGTATCCACGAG	RT-qPCR

MpACT-R	ACATGGTCGTTCCTCCAGAC	RT-qPCR
Mp1g20730-F	AGCTGACACTACGGTTCTGGT	RT-qPCR
Mp1g20730-R	GAGCTTCGACACATTTCACTGCC	RT-qPCR
Mp4g20750-F	GGCTGTACTACGTGCGTTTTCG	RT-qPCR
Mp4g20750-R	GACTGACGATATCTGTATGAATCG	RT-qPCR
Mp1g05970-F	CACTGCCAGCCCTTGTTGATA	RT-qPCR
Mp1g05970-R	TGTGGAGGAATGCTGCTTTCA	RT-qPCR
Mp1g15010-F	GCTTCGAGTCGTCCTTCATCA	RT-qPCR
Mp1g15010-R	GCTCCGGGATCAAAGCCAGCT	RT-qPCR
Mp6g13460-F	GTAGTGAGTCTGGCCTAGTGG	RT-qPCR
Mp6g13460-R	TCTACACAGTGGTCACAAGGG	RT-qPCR
miR160 probe	TGGCATACAGGGAGCCAGGCA	Northern blot
miR166 probe	GGGAATGAAGCCTGGTCCGAA	Northern blot
miR319ab probe	GGGAGCTCCCTTCAGTCCAAG	Northern blot
miR390 probe	GACGCTATCCCTCCTGAGCTT	Northern blot
miR408ab probe	AGCCAGGGAAGAGGCAGTGCA	Northern blot
miR156a/529 probe	GTGCTCACTCTCTTCTGTCA	Northern blot
miR1030 probe	GGTGCAGGTGCAGATGCAGA	Northern blot
MpmiR11737ab probe	GAGATTGTTTTCTTCCACGGGG	Northern blot
MpmiR11865* probe	ATGCATCTCCTCTGTGAAGCA	Northern blot
MpmiR11887 probe	GAGCTTAGCCTAGACACAAAC	Northern blot
MpmiR11796 probe	AGATCCCACAAATCGACTGGA	Northern blot
U6 probe	TCATCCTTGCGCAGGGGCCA	Northern blot
